# In vivo noninvasive mitochondrial redox assessment of the optic nerve head to predict disease

**DOI:** 10.1093/pnasnexus/pgad148

**Published:** 2023-05-02

**Authors:** Bertan Cakir, Yohei Tomita, Hitomi Yagi, Padraic Romfh, William Allen, Minji Ko, Peili Chen, Zhongjie Fu, Daryoosh Vakhshoori, Lois E H Smith

**Affiliations:** Department of Ophthalmology, Boston Children’s Hospital, Harvard Medical School, Boston, MA 02115, USA; Department of Ophthalmology, Boston Children’s Hospital, Harvard Medical School, Boston, MA 02115, USA; Department of Ophthalmology, Boston Children’s Hospital, Harvard Medical School, Boston, MA 02115, USA; Pendar Technologies, Cambridge, MA 02138, USA; Department of Ophthalmology, Boston Children’s Hospital, Harvard Medical School, Boston, MA 02115, USA; Department of Ophthalmology, Boston Children’s Hospital, Harvard Medical School, Boston, MA 02115, USA; Pendar Technologies, Cambridge, MA 02138, USA; Department of Ophthalmology, Boston Children’s Hospital, Harvard Medical School, Boston, MA 02115, USA; Pendar Technologies, Cambridge, MA 02138, USA; Department of Ophthalmology, Boston Children’s Hospital, Harvard Medical School, Boston, MA 02115, USA

**Keywords:** resonance Raman spectroscopy, glaucoma, mitochondria, metabolism, redox biology

## Abstract

Eye diseases are diagnosed by visualizing often irreversible structural changes occurring late in disease progression, such as retinal ganglion cell loss in glaucoma. The retina and optic nerve head have high mitochondrial energy need. Early mitochondrial/energetics dysfunction may predict vulnerability to permanent structural changes. In the in vivo murine eye, we used light-based resonance Raman spectroscopy (RRS) to assess noninvasively the redox states of mitochondria and hemoglobin which reflect availability of electron donors (fuel) and acceptors (oxygen). As proof of principle, we demonstrated that the mitochondrial redox state at the optic nerve head correlates with later retinal ganglion loss after acute intraocular pressure (IOP) elevation. This technology can potentially map the metabolic health of eye tissue in vivo complementary to optical coherence tomography, defining structural changes. Early detection (and normalization) of mitochondrial dysfunction before irreversible damage could lead to prevention of permanent neural loss.

## Introduction

The retina and the unmyelinated axons of retinal ganglion cells at the optic nerve head are highly metabolically active. Mitochondrial dysfunction contributes to age-related macular degeneration, diabetic retinopathy, and glaucoma (1, 2). Disease assessment is based on documenting irreversible late-stage structural changes with fundus photography and optical coherence tomography. It is crucial to detect (and treat) early cellular dysfunction before irreversible morphologic and functional vision changes occur.

We developed a technology using resonance Raman spectroscopy which noninvasively quantifies in vivo and in real time the redox states of mitochondrial cytochrome complexes and of hemoglobin (3, 4) in murine eyes. The majority of energy in the retina and optic nerve head is generated by mitochondrial oxidative phosphorylation and aerobic glycolysis (5, 6). In the mitochondria, energy from carbohydrates, fatty acids, and amino acids is translated into high-energy electron bonds in NADH and FADH_2_. The electrons pass through the electron transport chain via mitochondrial cytochromes to the final electron acceptor, oxygen, releasing energy in the process which generates a proton gradient driving ATP production. Therefore, the relationship between reduced and oxidized cytochromes in the mitochondria reflects the balance of fuel (electron donors) and oxygen (electron acceptor).

Glaucoma is a leading cause of blindness globally (7). Mitochondrial dysfunction is important in disease pathogenesis (8–17). Elevated intraocular pressure (IOP) is highly associated with progressive loss of retinal ganglion cell (RGC); lowering IOP is the only current treatment to prevent ganglion cell loss.

Early detection of mitochondrial dysfunction with real-time mitochondrial redox state assessment could lead to early intervention prior to irreversible damage in glaucoma or other retinal disorders.

## Results and discussion

**Fig. 1.**
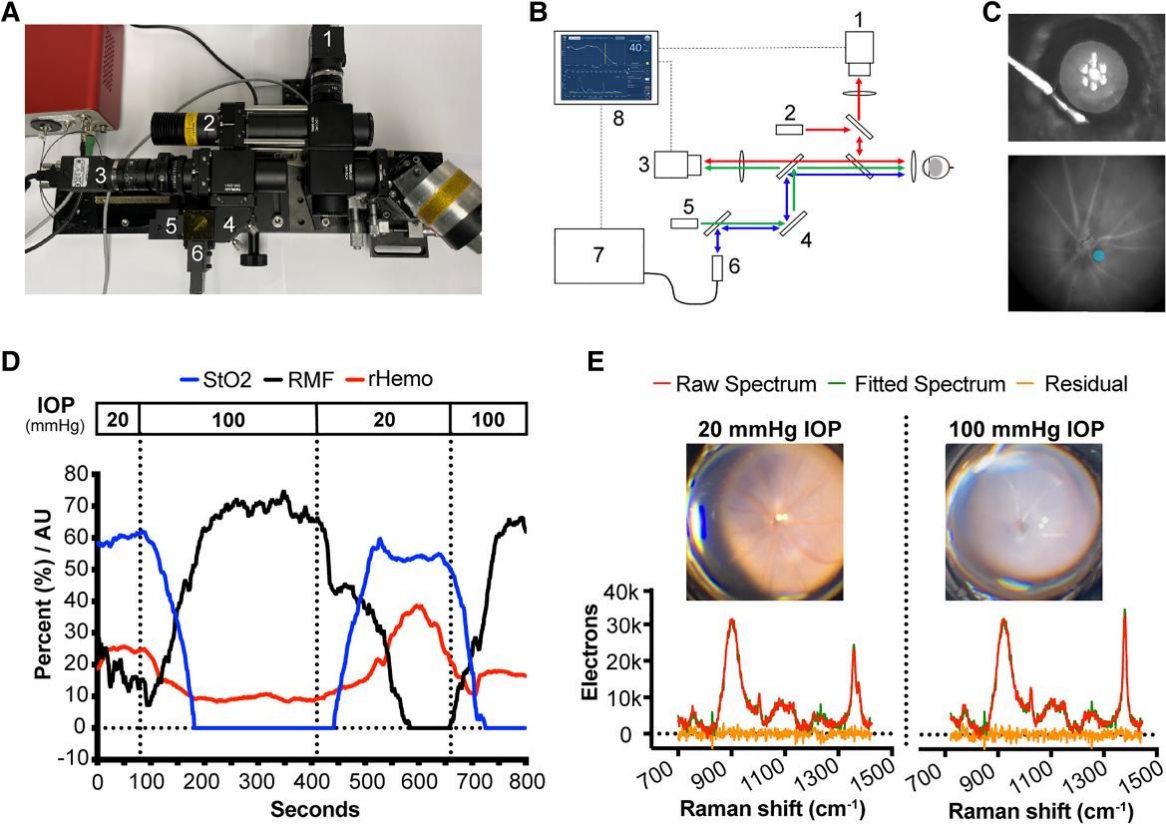
In vivo resonance Raman redox state assessment of mitochondrial cytochromes and hemoglobin in a mouse model of elevated IOP. A, B) The eye imager (and schematic) with integrated Raman spectrometer: 1) anterior segment camera, 2) fundus illumination light source, 3) fundus camera, 4) mirror to position light on the fundus, 5) aiming laser, 6) Raman probe head, 7) spectrometer, and 8) computer with LabView control software. C) Top: anterior chamber cannulation. The cannula is connected to a fluid reservoir to allow IOP manipulation by height changes of the fluid reservoir. Bottom: fundus imaging (circle = area of measurement). D) StO_2_ fraction (in %; blue line), RMF (in %; black line), and hemoglobin signal strength (rHemo in AU; red line) in response to dynamic IOP changes alternating between 20 and 100 mmHg (averaged over 120 s). E) Raman spectrum and fundus images at 20 (left) and 100 mmHg (right). Raw spectrum after baseline adjustment (red line); fitted spectrum with available spectral library (green line); and residual unexplained spectrum (orange line). The spectrum is well explained with the residual being close to shot-noise limit. A significant shift of the ν_4_ peak (1,350–1,375cm^−1^) is noted when the IOP is elevated to 100 mmHg.

To demonstrate the potential of a noninvasive technique to assess ocular redox states of mitochondria and hemoglobin to assess future RGC loss, we utilized a well-described retinal ischemia–reperfusion mouse model. We varied the IOP and correlated the redox states of mitochondrial cytochromes and hemoglobin at the optic nerve head with later RGC loss (Fig. 1A–C).

**Fig. 2.**
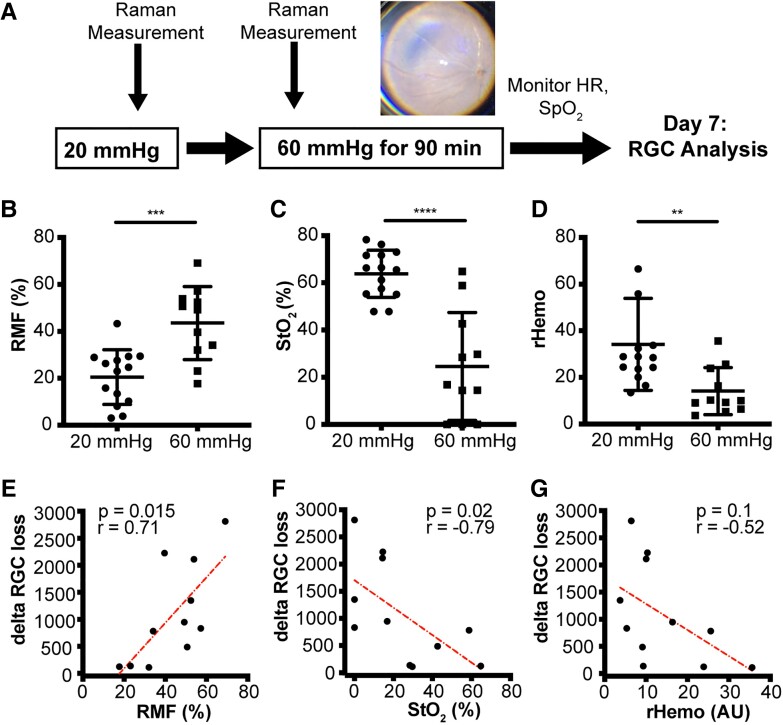
RMF correlates with RGC loss. A) Schematic of experimental paradigm with fundus image at 60 mmHg. B) RMF increases more than 2 times at 60 mmHg IOP versus baseline (*n* = 14 and 11, *P* = 0.0003) C). StO_2_ fraction decreases more than 2 times after IOP elevation to 60 mmHg (*n* = 14 and 11, *P* < 0.0001). D) rHemo (total hemoglobin) is decreased at 60 mmHg IOP compared with baseline (*n* = 14 and 11, *P* = 0.0056). E) RMF at 60 mmHg correlates with ganglion cell (RGC) loss at day 7 (*n* = 11, *r* = 0.7, *P* = 0.015). F) Hemoglobin oxygenation fraction correlates negatively with RGC loss at day 7 (*n* = 11, *r* = −0.62, *P* = 0.042). G) rHemo did not correlate with RGC degeneration (*n* = 11, *r* = −0.52, *P* = 0.1). B–D) Unpaired *t* test used for statistical analysis. Data presented as mean ± SD. **P < 0.01, ***P < 0.001, and ****P < 0.0001. E–G) Pearson correlation coefficient was used for statistical analysis.

IOPs at 100 mmHg (near the mean arterial pressure in mice) (18) resulted in a rapid increase in reduced mitochondrial fraction (RMF), a decrease in blood oxygenation, and a reduction in total Raman hemoglobin signal strength (Fig. 1D). This constellation is consistent with IOP-induced hypoperfusion resulting in an insufficient supply of oxygen (the final electron acceptor) leading to a backup of electrons in the electron transport chain with an increase in the reduced mitochondrial cytochrome fraction. This is supported by the visible whitening of the fundus image (Fig. 1E) and the reduction of the hemoglobin signal strength by more than half after IOP elevation (Fig. 2D). With reduction of the IOP back to 20 mmHg, the signals returned to baseline. Interestingly, a transient peak in total hemoglobin strength was observed which possibly represents increased reperfusion after hypoperfusion.

At 60 mmHg IOP, we found a wide variation of RGC degeneration (Fig. S1) suggesting a threshold for ischemia and ganglion cell damage. IOP of 60 mmHg led to an increase in RMF (*P* = 0.0003) and a decrease in both oxygenated hemoglobin fraction (*P* < 0.0001) and total hemoglobin signal strength (rHemo) (*P* = 0.0015) compared with baseline (Fig. 2B–D). This is likely due to a range of tissue ischemia given the reduced total hemoglobin signal and partial compression of the vasculature (Fig. 2A). More importantly, there was a linear correlation between the RMF and RGC loss (*r* = −0.71, *P* = 0.015; Fig. 2E). The oxygenated hemoglobin fraction also correlated but negatively (*r* = −0.62, *P* = 0.042; Fig. 2F), suggesting that the degree of ischemia impacts future RGC degeneration.

We have previously shown that in an ischemic heart rat model, an increase in the reduced mitochondrial ratio tightly correlated with tissue oxygen tension (19). Assessing the oxygen content and the redox state of the oxygen sink (mitochondria) simultaneously as shown in this work could allow the assessment of mitochondrial dysfunction other than that induced by ischemia. In this study, we have not explored the utility of redox state assessment other than ischemia-induced ganglion cell loss. To our knowledge, there is no published study examining other cellular dysfunction using resonance Raman spectroscopy in vivo. Ex vivo experiments, however, have shown that Raman scattering is sensitive to changes in mitochondrial redox states, mitochondrial membrane potential, activity of the electron transport chain, and mitochondrial viability which are the core elements of mitochondrial function (20–22). These ex vivo studies are promising indicators for the potential of resonance Raman spectroscopy to determine mitochondrial dysfunction in vivo caused by pathology other than ischemia.

The strength of resonance Raman spectroscopy is that it can determine relative quantities of both reduced and oxidized forms of heme containing structures to assess reduced or oxidized fractions. Determining the fractions has an advantage over assessing absolute signal intensities since the intensity of a signal is dependent on media clarity (affected by cataracts, etc.) which can vary greatly from eye to eye, particularly in older patients, the group most commonly susceptible to disease.

The downside of this technology is that Raman scattering is a relatively weak phenomenon which requires relatively higher excitation energies to obtain a low signal-to-noise ratio. We used a 430-nm single-mode laser with an excitation energy of 4 mW with an exposure time of 120–180 s to achieve sufficient data collection. Particularly short-wavelength lasers are potentially harmful to the human retina, so improving efficiency of the system will be necessary to achieve safe laser exposure levels in the human eye according to the American Standard Institute (ANSI). In our mouse model, there was no significant RGC degeneration in either laser-exposed or nonlaser-exposed controls (Fig. S2). Further, the animal model used in this work poses several challenges. One limitation is the rapid cataract formation in anesthetized mice only allowing robust data collection in the first 20–30 min. This limited us from doing longitudinal data analysis throughout the 90-min IOP elevation. Another challenge in mice is the small nature of anatomical structures. Due to the small, crowded optic nerve head with large venous and arterial vessels entering and exiting the eye, even minor positional changes can lead to significant variations in heme redox value readings. This, in part, likely explains some of the spread seen in our data. To avoid this variation as much as possible, we took great care to aim the beam in between the large vessels while trying to stay on the optic nerve head. This resulted in some instances the excitation beam to be partially positioned on the peripapillary region.

In summary, our study has established proof of concept of simultaneous redox state assessment of ocular mitochondria and hemoglobin in vivo in real time and shows correlation with RGC degeneration in an acute IOP model using resonance Raman spectroscopy.

## Materials and methods

We integrated a retinal imaging system with our resonance Raman spectroscopy cellular energetics device with a low-power (4 mW) 430-nm laser source (Fig. 1A and B) (19). A Raman spectrum was captured from the optic nerve head each second and averaged over 180 s, and the RMF and the tissue oxygenated hemoglobin (StO_2_) ratio was calculated (19). The rHemo in arbitrary units (AU) value reflects the resonance Raman spectrum explained by hemoglobin, which changes proportionally with total tissue blood. The anterior chamber was connected to a fluid reservoir to assess the Raman spectrum at the optic nerve head under varying IOPs (Fig. 1D and E). RGC counts were analyzed at day 7 and correlated with the Raman measurements.

A detailed description of Materials and Methods can be found in Supplementary material.

## Supplementary Material

pgad148_Supplementary_Data

## Data Availability

All study data are included in the article and/or Supplementary material.
