# Supplementary material for: In vivo noninvasive mitochondrial redox assessment of the optic nerve head to predict disease
Source: PNAS Nexus. 2023 May 2;2(5):pgad148. doi: 10.1093/pnasnexus/pgad148 (PMC10230116; doi:10.1093/pnasnexus/pgad148)
Supplement: pgad148_Supplementary_Data [file pgad148_supplementary_data.zip › PNASNEXUS-PNASNEXUS-2022-01295-T-s01.docx]

**Supporting Information for**

**In vivo non-invasive mitochondrial redox assessment of the optic nerve head to predict disease**

Bertan Cakir^1*,^ ^†^, Yohei Tomita^1*^, Hitomi Yagi^1^, Padraic Romfh^2^, William Allen^1^, Minji Ko, Peili Chen^2^, Zhongjie Fu^1^, Daryoosh Vakhshoori^2^, Lois E. H. Smith^1†^

**^†^Corresponding Authors:**

Lois E.H. Smith, MD, PhD

Institution: Boston Children’s Hospital/ Harvard Medical School

Address: Children's Hospital, 300 Longwood Ave, Boston, MA 02115

Phone number: +1-617-919-2529, Email: [Lois.Smith@childrens.harvard.edu](mailto:Lois.Smith@childrens.harvard.edu)

Bertan Cakir, MD

Institution: Boston Children’s Hospital/ Harvard Medical School

Address: Children's Hospital, 300 Longwood Ave, Boston, MA 02115

Phone number: +1-857-352-8361Email: [Cakir.Bertan@childrens.harvard.edu](mailto:Cakir.Bertan@childrens.harvard.edu)

**This PDF file includes:**

Supporting text: Extended Methods and Materials

Supporting Information Text

**Extended** **Materials and Methods**

*Device description*

We designed and built an integrated retinal imaging system optimized for use with our existing resonance Raman spectroscopy cellular energetics device (Fig 1A & B). Briefly, as previously described, the Raman spectroscopy cellular energetics device couples a single-line, low-power (4 mW) 430nm laser source to an optical fiber which carries it to a probe head from which it is directed to the surface of the target tissue (1). Inelastically scattered light (resonance Raman scattering) is captured by the probe optics and returned in a second optical fiber to the spectrometer, where it is passed through a grating onto a high-resolution charged-coupled device (CCD). The CCD is read once per second by our custom software and a time-averaged spectrum analyzed to determine the relative contribution of chromophore spectra from a stored library. The retinal imaging system can visualize the retina with sufficient detail to aim the resonance Raman spectroscopy pump beam on the optic nerve head and avoid structures such as major blood vessels. At the surface of the cornea, the beam diameter is 1.5 mm, which is focused to an approximately 50μm spot on the retina by the mouse eye lens which is sufficiently small to target the beam between major vessels on the optic nerve head of the mouse. The mouse is positioned in a temperature controlled conical holder that steadies the eye and allows alignment at the front lens. There are two paths, separated by the primary beam splitter. The alignment path used to align the system to the eye consists of an LED light source and the pupil camera coupled through the alignment beam splitter. The laser light from the resonance Raman spectroscopy probe is coupled with an aiming laser, and then coupled through the measurement beam splitter into the measurement path and finally into the primary path to the eye. The retina camera is positioned with a focusing lens through the measurement beam splitter and into the primary path. Because the resonance Raman spectroscopy excitation laser and the Raman scattering were in the <500 nm range, light above this wavelength is passed to the retina camera and the 430 nm resonance Raman spectroscopy laser is not visible in the retina camera. The 532 nm aiming laser passes through the measurement path, but is returned to the retina camera, allowing visualization of the laser position for fine adjustments of the target location.

The cameras and the resonance Raman spectroscopy system are controlled using custom developed Labview software running on an attached computer. Camera images can be viewed live. The laser and spectrometer are controlled by the software, with spectral data collected from the CCD passed into the software for analysis using the regression algorithm as previously described (1).

*Resonance Raman spectrum analysis*

Individual chromophores, including the oxidized and reduced states of the mitochondrial cytochrome complexes as well as the oxidized and reduced forms of hemoglobin, exhibit maximum resonant enhancement when excited near their Soret absorption peak. For the targets of this study, the absorption peak occurs between 400 to 450 nm. By using a 430 nm excitation source, we achieved a balanced spectrum with contributions from each of the target chromophores.

Quantification of any substance using Raman spectroscopy requires a wavelength-specific library against which a sample reading can be compared and regressed. A Raman spectral library of heme containing structures such as mitochondrial complexes and hemoglobin was established in our prior work in the heart (1). To further complement the Raman spectral library for in vivo measurements in the eye we added wavelength specific Raman spectra of each mouse eye component to the library. First, each component of the eye was dissected separately and measured using our RRS device to generate a spectral library of the eye components. We determined that only the mouse eye lens was contributing significantly to the resonance Raman spectroscopy spectrum, so this library was used for further analysis. This spectral library was then utilized to quantify the heme containing structures from the collected resonance Raman spectrum using a regression algorithm as previously described (1). This allowed the calculation of the reduced mitochondrial fraction (RMF) and the tissue oxygenated hemoglobin ratio (StO2). The total hemoglobin signal strength (rHemo) is quantified in arbitrary units (AU) and is based on the sum of the regression coefficients (CCD electrons) for reduced and oxidized hemoglobin divided by the laser power (mW). The rHemo value reflects the amount of the resonance Raman spectrum that is explained by hemoglobin, and it will change proportionally with the amount of blood in the tissue.

*Modified Ischemic reperfusion model*

Adult mice were anesthetized by inhalation of isoflurane (2-4%) in the induction chamber of the Somnosuite low-flow anesthesia system (Kent Scientific Corporation, Torrington CT, USA). Animals were then transferred to nose cone inhalation with 1-1.5% isoflurane for the duration of the study. Mice were kept on temperature-controlled surfaces during anesthesia and throughout the procedure to maintain adequate body temperature. Under the surgical microscope, a 33-gauge cannula needle was inserted into the anterior chamber. The contralateral eye served as an untreated control. A fluid reservoir (BSS Plus, Alcon, Fort Worth, Texas) was held at various heights to increase the intra-ocular pressure. Mice were excluded if any leakage from the cannulation site was observed, or significant structural damage was noted to the iris or lens. Artificial tears (Isoptotears) were applied to lubricate the eye before placement of a 0-diopter contact lens on the cannulated eye (OOPS Inc., Japan). The mouse was placed on a temperature controlled adjustable mouse stage to align the mouse eye with the Raman-Spectroscopy system. The intraocular pressure was validated using a tonometer (TonoLab; Colonial Medical Supply, Espoo, Finland). During the experiment the heart rate and blood oxygenation of the mouse were monitored using pulsoxymetry on the paw (Kent Scientific Corporation, Torrington CT, USA). The blood oxygenation was targeted to be above 80% and mice were excluded if the measurement dropped below that threshold. At the end of the experiment, the needle was removed, ointment was placed on the corneal surface and the mouse was placed on a heating pad to recover from anesthesia. The mice were sacrificed, and eyes enucleated seven days after the procedure for immunohistochemical staining of retinal ganglion cells.

*Immunohistochemistry for Retinal Ganglion Cells*

The procedure for immunohistochemistry was as previously described (2). Briefly, eyes were enucleated and fixed in 4% PFA for 2 hours. The retina was whole-mounted, and four radial cuts were made to flatten the tissue. Then, the retina was blocked with Mojito Buffer overnight at 4°C with shaking. The retina was washed in Washing Buffer at room temperature and incubated in anti-Brn3a (Millipore MAB1585, Mouse, 1:500) diluted in Staining Buffer overnight at 4°C with shaking. Then the retina was washed in Washing Buffer at room temperature and incubated in a secondary antibody (Biotinylated Anti-mouse antibody, BA-9200, 1:250, Invitrogen, Carlsbad, CA) diluted in Staining Buffer for 2 hours at room temperature. The retina was washed in Washing Buffer at room temperature. Then retina was incubated with the tertiary antibody (Alexa-Streptavidin, Invitrogen, S32354, 1:400) diluted in Staining Buffer for 1 hour at room temperature and was covered with foil to avoid light. Finally, the retina was washed in Washing Buffer at room temperature (avoiding light). The retina was mounted on a glass slide with mounting media.

*Study Approval*

All animal studies were in compliance to the Association for Research in Vision and Ophthalmology Statement for the Use of Animals in Ophthalmic and Vision Research and were approved by the Institutional Animal Care and Use Committee at Boston Children’s Hospital. Wild-type C57BL/6J mice were obtained from the Jackson Laboratory (Bar Harbor, ME).

*Statistics*

All data are presented as the mean ± SEM. A two-tailed unpaired t-test was used to compare the results (Prism v9.0; GraphPad Software, Inc., San Diego, CA). The threshold for statistical significance (*α*) was set at 0.05.

**Supplemental Figures**

**Supplemental Figure 1.**

Retinal ganglion cell (RGC) count in control mice and mice with intraocular pressure (IOP) elevation to 60 mmHg for 90 min on day 7. A large variation in RGC loss was seen in 60 mmHg group.

**Supplemental Figure 2.**

Retinal ganglion cell (RGC) count in control mice and mice exposed to the 4mW 430nm excitation laser without intraocular pressure elevation. No significant difference in RGC count was seen between both groups.

**SI References**

1. D. A. Perry, *et al.*, Responsive monitoring of mitochondrial redox states in heart muscle predicts impending cardiac arrest. *Sci. Transl. Med.* **9** (2017).

2. Z. Xu, *et al.*, Neuroprotective role of Nrf2 for retinal ganglion cells in ischemia-reperfusion. *J. Neurochem.* **133**, 233–241 (2015).
