# Supplementary figures and images for: In vivo noninvasive mitochondrial redox assessment of the optic nerve head to predict disease
Source: PNAS Nexus. 2023 May 2;2(5):pgad148. doi: 10.1093/pnasnexus/pgad148 (PMC10230116; doi:10.1093/pnasnexus/pgad148)

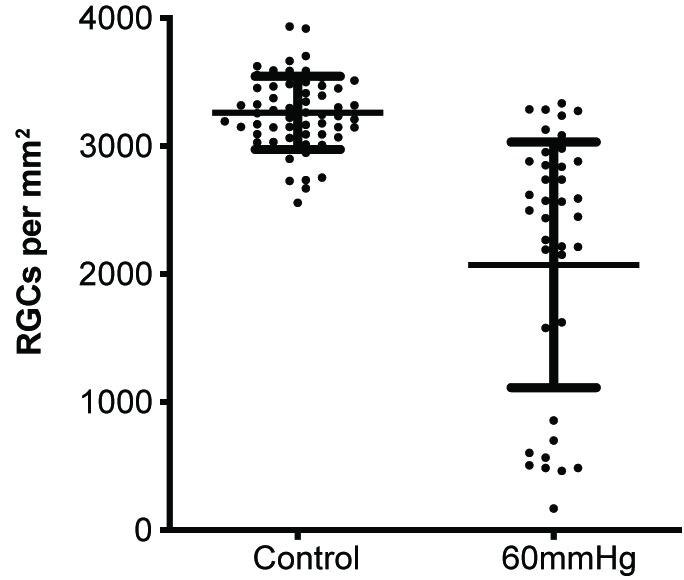

Supplement: pgad148_Supplementary_Data [file pgad148_supplementary_data.zip › PNASNEXUS-PNASNEXUS-2022-01295-T-s02.tif]

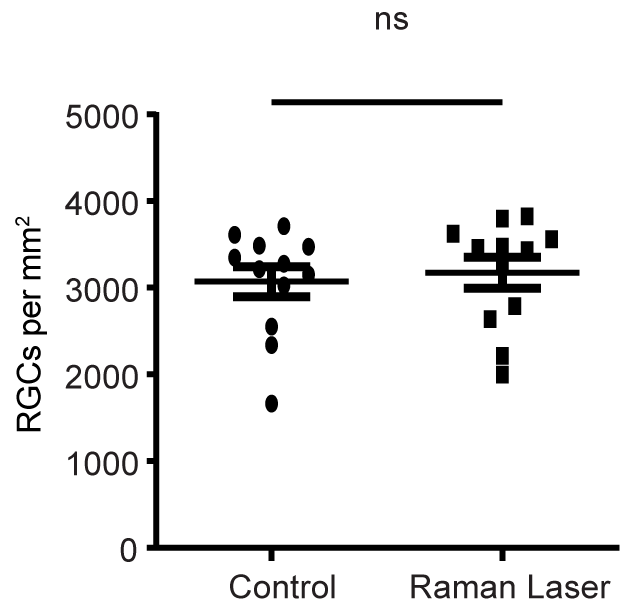

Supplement: pgad148_Supplementary_Data [file pgad148_supplementary_data.zip › PNASNEXUS-PNASNEXUS-2022-01295-T-s03.tif]
